# Letter to the editor: pre-emptive infiltration with betamethasone and ropivacaine for postoperative pain in laminoplasty and laminectomy (PRE-EASE): a prospective randomized controlled trial

**DOI:** 10.1097/JS9.0000000000001256

**Published:** 2024-03-04

**Authors:** Peng Ye, Xuan Pan, Ting Zheng, Xiaochun Zheng

**Affiliations:** a Department of Anesthesiology, Shengli Clinical Medical College of Fujian Medical University, Fujian Provincial Hospital; b Fujian Emergency Medical Center, Fujian Provincial Key Laboratory of Emergency Medicine, Fujian Provincial Key Laboratory of Critical Care Medicine, Fujian Provincial Co-Constructed Laboratory of “Belt and Road”, Fuzhou, People’s Republic of China

*Dear Editor*,

We have read a recent article titled ‘Pre-emptive infiltration with betamethasone and ropivacaine for postoperative pain in laminoplasty and laminectomy (PRE-EASE): a prospective randomized controlled trial^1’^, with great interest. This study presents valuable findings that pre-emptive infiltration with a betamethasone and ropivacaine mixture can effectively reduce postoperative pain and opioid consumption following spine surgeries. While the authors have acknowledged the limitations in the discussion section, there are still some unanswered questions within the article that we would appreciate the authors’ insights and comments on.

First, ultrasound guidance was not employed during the drug injection. Although no serious complications have been observed, research has indicated that a combination of betamethasone and ropivacaine may lead to crystallization^2^. In the spine’s blood supply rich region, this could increase the risk of the local anesthetic inadvertently entering the blood vessels and epidural space, potentially raising the likelihood of embolism^3^. Therefore, we recommend thorough aspiration checks during injection or the use of ultrasound equipment to enhance the safety of research.

Second, the study presents the finding that ‘The visual analog scale (VAS) scores at movement and rest were also significantly lower until 3 months and 6 weeks, respectively’. Nevertheless, prior research has demonstrated that a medical history of chronic pain and the number of cervical levels significantly affect postoperative pain^4^. Thus, to enhance the informative value of the conclusions, it is recommended that a stratified analysis be conducted based on the past medical history of chronic pain and number of levels treated cervical level of patients. Additionally, it needs to be clarified whether the surgeries were performed by the same group of surgeons, as different surgical techniques from different surgeons could potentially affect patients’ postoperative pain.

In conclusion, I commend the authors’ contributions to the field of postoperative pain management. Addressing these points offers a pathway to not only enhance the current study but also to guide future research towards comprehensive, patient-centered approaches for managing pain after surgical interventions.

## Ethical approval

This article does not require any human or animal subjects to acquire such approval.

## Consent

This article does not require any human or animal subjects to acquire such approval.

## Funding information

The authors received no financial support.

## Author contribution

X.Z.: conceptualized and review and editing; P.Y., X.P., and T.Z.: writing, made the draft, and updated it. All authors have critically reviewed and approved the final draft and are responsible for the content and similarity index of the manuscript.

## Conflicts of interest disclosure

The authors declares no competing interest.

## Research registration unique identifying number (UIN)

None.

## Guarantor

All authors.

## Data availability statement

The datasets utilized and/or analyzed in this study can be obtained from the corresponding author upon a reasonable request. Data availability statement: This study did not have any data analysis.

## Provenance and peer review

Not commissioned, externally peer-reviewed.
